# Cross-Cohort Transcriptomic Prioritization and Chemical-Provenance Assessment of Asthma Airway-Brushing Genes Overlapping HERB-Annotated *Scrophularia ningpoensis* Targets

**DOI:** 10.3390/ijms27177886

**Published:** 2026-09-03

**Authors:** Jinhao Zou, Xiaowei Tian, Siyi Wang, Ye Sun

**Affiliations:** 1 Key Laboratory of Dryness Syndrome in Chinese Medicine, Ministry of Education, Ningxia Medical University, Yinchuan 750004, China; pullenda20@gmail.com; 2 College of Traditional Chinese Medicine, Ningxia Medical University, Yinchuan 750004, China; 18295171979@163.com; 3 College of Pharmacy, Ningxia Medical University, Yinchuan 750004, China

**Keywords:** *Scrophularia ningpoensis*, asthma, airway brushing, transcriptomics, molecular informatics, cross-cohort transferability, chemical identity normalization, evidence provenance

## Abstract

Direct human evidence linking *Scrophularia ningpoensis* to asthma is limited, and database-derived herb–target records do not establish constituent exposure or target engagement. We used an evidence-audited molecular-informatics workflow to prioritize asthma airway-brushing-associated genes overlapping a frozen HERB export. GSE63142 was the discovery set, GSE67472 the independent airway-epithelial replication set, GSE137268 an induced-sputum cross-biospecimen transfer set, and GSE43696 a descriptive same-cohort reference because 105 of 108 identifiers overlapped GSE63142. Of 1266 measurable HERB-annotated targets, an 81-gene FDR-defined overlap did not exceed an expression-matched null (*p* = 0.0972), whereas a 19-gene strict overlap showed 2.27-fold enrichment (empirical *p* = 3.00 × 10^−4^) and 12 genes met the prespecified replication criterion in GSE67472. Provenance sensitivity retained two genes after database-mining-only edges were excluded and none under a direct-target-engagement requirement. Nested classifiers transferred to GSE67472, but not to sputum. Out-of-fold SHAP identified model-specific contributors, with moderate cross-cohort rank stability for LASSO (Spearman ρ = 0.563) and random forest (ρ = 0.693); SHAP was not interpreted as causal or herbal-target importance. These findings support computational prioritization and transferability assessment, not chemical presence, exposure, target binding, mechanism, or efficacy.

## 1. Introduction

Asthma is a heterogeneous airway disease whose clinical phenotypes reflect partly distinct molecular and inflammatory patterns [1,2]. The airway epithelium contributes to barrier regulation, immune sensing, remodeling, and treatment-responsive transcriptional programs [3,4]. Airway brushings therefore provide disease-relevant transcriptomic material, but they remain bulk specimens; measured expression can reflect epithelial states together with variable immune or stromal admixture. Interpretation also depends on whether datasets are genuinely independent and whether apparent signals persist across platforms and biospecimens.

Scrophulariae Radix (Xuanshen) is the dried root of *Scrophularia ningpoensis* Hemsl. and contains iridoid glycosides, phenylpropanoid glycosides, and other constituents [5]. Chemical studies have reported eugenol, ferulic acid, β-sitosterol, adenosine, aucubin, harpagoside, acteoside, daucosterol, succinic acid, ursolic acid, and additional glycosides in previously examined roots [6,7,8,9,10,11,12,13,14,15]. Identification strength varies from isolation or authentic-standard confirmation to tentative mass-spectrometric assignment [10,11,12,13,14,15,16]. These reports establish only prior occurrence in the analyzed root materials. The present study did not examine a botanical sample or preparation. Moreover, direct human evidence connecting *S. ningpoensis* with asthma is limited; experiments involving other *Scrophularia* species or animal allergic-airway models [17,18,19,20] provide context but cannot establish efficacy or a mechanism for *S. ningpoensis* in human asthma.

Network pharmacology provides a systems framework for examining multicomponent and multitarget hypotheses [21,22], but traditional-medicine databases differ in coverage, annotation rules, herb specificity, and evidence provenance [23]. Intersections between disease-associated genes and database-derived herb targets may be inflated by shared targets, chemical aliases, or broad database-mining associations. Large intersections can occur by chance, and enrichment results depend on the background universe [24,25]. Stable chemical identifiers can reduce alias duplication [26], but neither identity normalization nor prior botanical occurrence demonstrates that a constituent was present in a study preparation, reached the airway, or engaged a recorded target. Transcriptomic validation has parallel vulnerabilities: separate GEO accessions can reuse participants, bulk airway brushings can contain variable cell mixtures, and model transfer can deteriorate under biological or technical shift [27]. Outcome-informed filtering before cross-validation can further overstate prediction [28,29].

The unresolved question was therefore not whether a database intersection could be converted into a herbal mechanism, but whether a defensible candidate set would remain after cohort-independence, overlap-null, chemical-identity, provenance, and transferability checks. We treated GSE63142 as discovery, GSE67472 as independent airway-epithelial replication, GSE43696 as a descriptive same-cohort reference after participant-overlap auditing, and GSE137268 as a cross-biospecimen transfer set. Differential expression, overlap testing, enrichment, STRING connectivity, and nested machine learning were kept analytically distinct. We also examined metadata availability, sputum phenotypic strata, the exclusion of four prominent inflammatory or immune-associated markers, ingredient sharing across herbs, and increasingly stringent HERB evidence layers. The resulting scope is computational candidate prioritization and cross-cohort transferability assessment, not pharmacological or causal validation.

## 2. Results

### 2.1. Dataset Roles and Cohort Overlap

**Figure 1:**
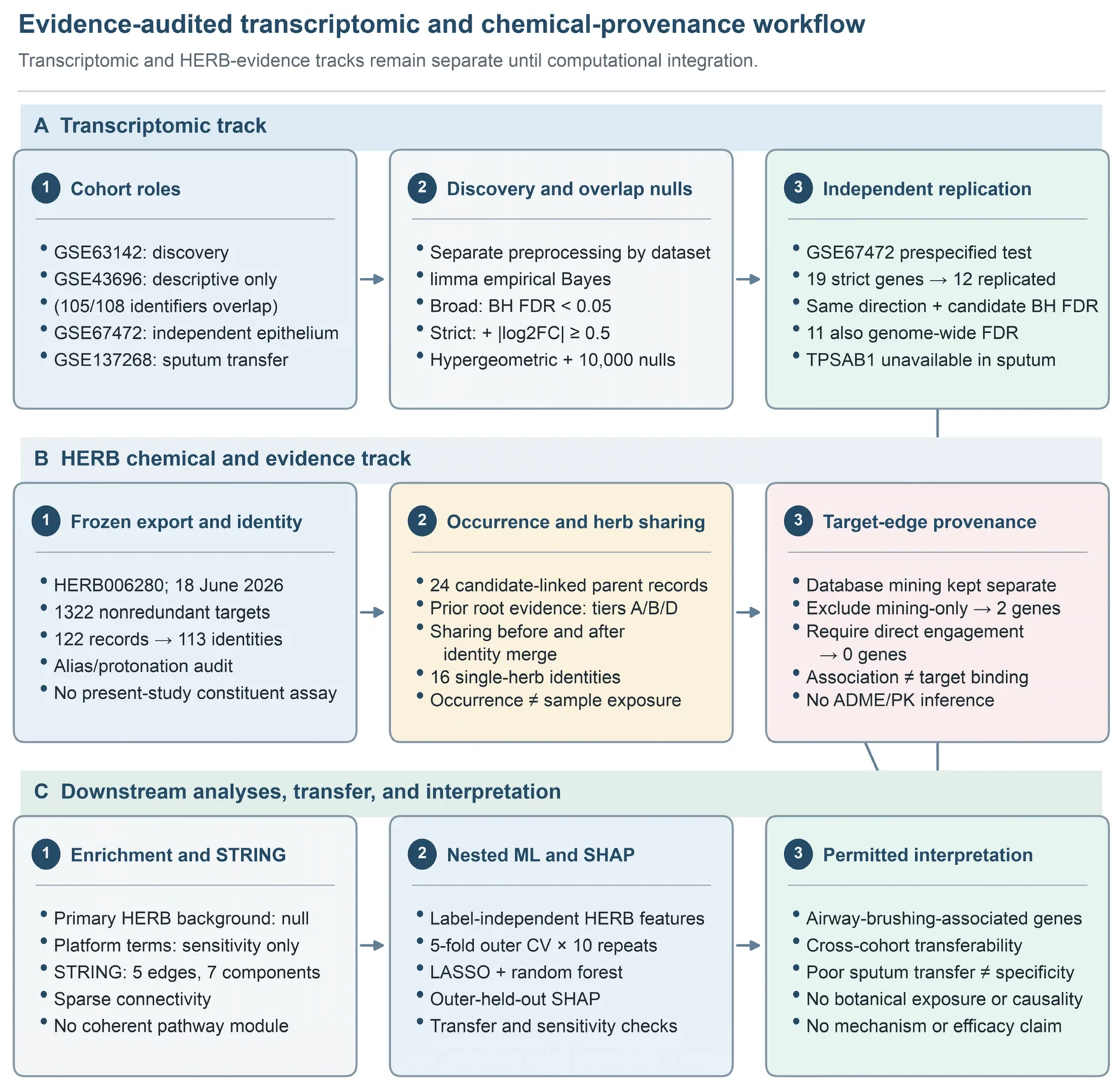
Evidence-audited workflow with explicit cohort roles and separated analytical tracks. The frozen HERB006280 export and four GEO datasets entered separate provenance and transcriptomic tracks. GSE63142 was used for discovery; GSE43696 was retained only as a descriptive same-cohort reference after 105 of 108 identifiers were found to overlap discovery; GSE67472 provided independent airway-epithelial replication and external model testing; and GSE137268 provided cross-biospecimen transfer and subgroup inspection. Differential expression, matched-null overlap testing, epithelial replication, chemical-identity and botanical-specificity audits, enrichment, STRING analysis, repeated nested machine learning, out-of-fold SHAP assessment, and sensitivity analyses were kept distinct. The workflow ends at computational prioritization and transferability assessment; it does not establish constituent presence, exposure, target engagement, mechanism, or efficacy.

The four datasets contained 437 samples (Table 1; Figure 1). GSE43696 initially appeared suitable for same-platform validation, but 105 of its 108 short subject identifiers were shared with GSE63142. It was therefore excluded from independent performance and direction-consistency analyses. GSE67472 was the independent airway-epithelial replication dataset, and GSE137268 was used only to examine transfer to induced sputum.

Metadata availability differed substantially. Harmonized age, sex, severity, phenotype, treatment, inhaled-corticosteroid exposure, and smoking fields were unavailable for GSE63142. GSE43696 included age, sex, and moderate/severe status; GSE67472 included age and sex; and GSE137268 included age, sex, clinical-control strata, and inflammatory phenotype for 54 of 69 samples. Treatment, inhaled-corticosteroid exposure, and smoking could not be harmonized across the four datasets. Uniform covariate adjustment was therefore infeasible, and missing fields were not imputed.

### 2.2. HERB–Target Overlap in the Discovery Dataset

**Figure 2:**
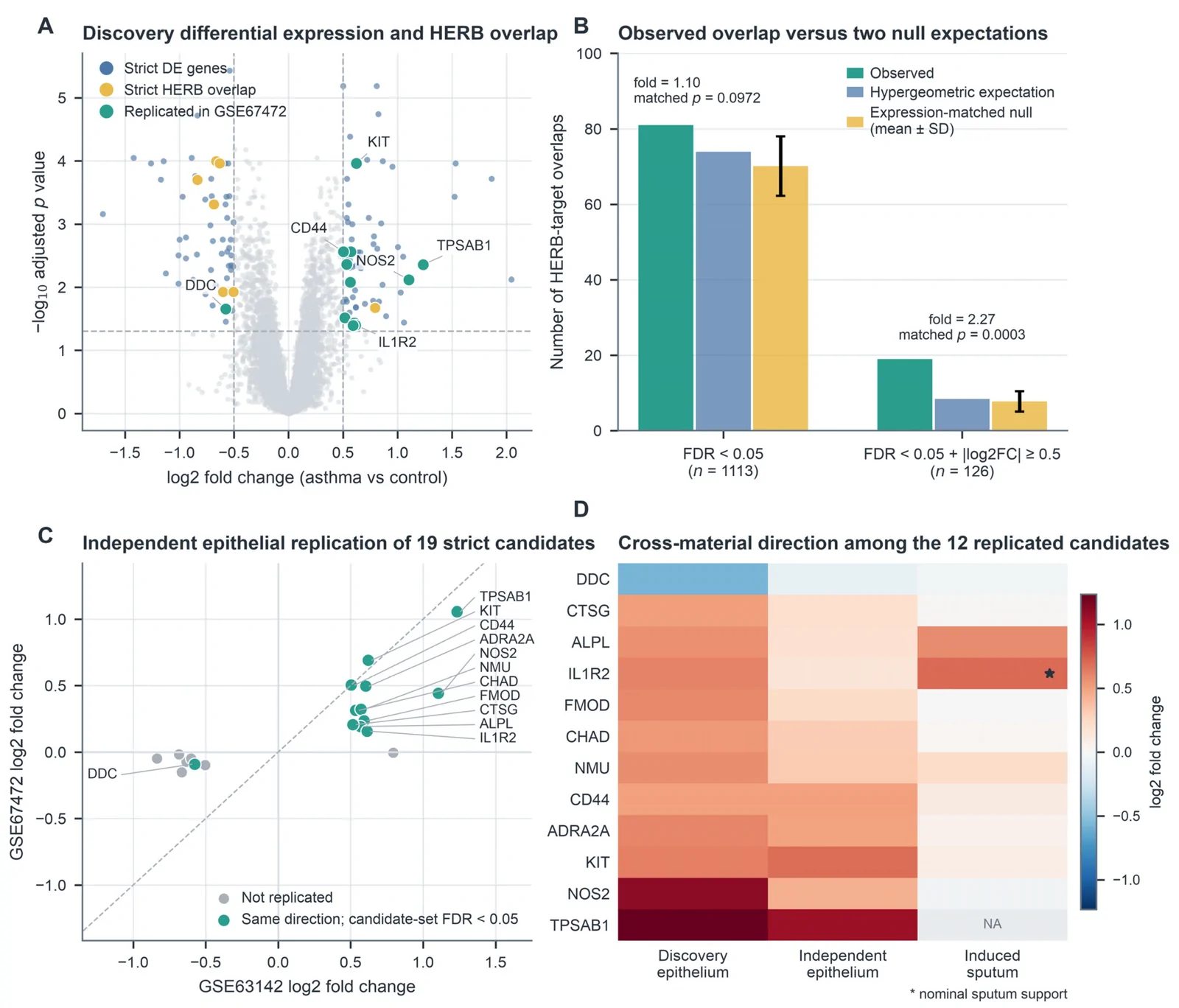
Discovery differential expression, HERB–target overlap, and independent airway-epithelial replication. (**A**) Limma volcano plot for GSE63142. Light-gray points denote genes outside the highlighted strict HERB-overlap categories, and gray dashed lines mark FDR = 0.05 and |log2FC| = 0.5. The 19 strict HERB-overlap candidates met FDR < 0.05 and |log2FC| ≥ 0.5; 12 also showed the same direction and candidate-set FDR < 0.05 in GSE67472. (**B**) Observed overlaps compared with the hypergeometric expectation and 10,000 expression-matched permutations. The FDR-only overlap was 81 genes versus 73.94 expected (1.10-fold; hypergeometric *p* = 0.206; empirical *p* = 0.0972), whereas the strict overlap was 19 genes versus 8.37 expected (2.27-fold; Fisher odds ratio 2.52; hypergeometric *p* = 6.49 × 10^−4^; empirical *p* = 3.00 × 10^−4^). (**C**) Effect-size comparison for the 19 strict candidates in GSE63142 and GSE67472. Gray points denote candidates that did not meet the replication rule, and the gray dashed diagonal is the y = x identity line. (**D**) Log2FC values for the 12 candidate-set replicated candidates across discovery airway brushing, independent airway epithelium, and induced sputum. NA denotes not available because no valid GPL6104 probe-to-gene mapping for TPSAB1 was present in either the GPL6104 platform annotation or the processed GSE137268 matrix; the star marks nominal sputum support for IL1R2. Sputum direction was not a replication criterion.

Limma identified 1113 of 19,057 discovery genes at BH FDR < 0.05, of which 126 also had |log2FC| ≥ 0.5 (Figure 2A). Eighty-one of the 1266 measurable HERB targets intersected the broad FDR set. The expected overlap was 73.94 (1.10-fold); the hypergeometric *p* was 0.206 and the expression-matched empirical *p* was 0.0972. Thus, the broad intersection did not exceed the matched null (Figure 2B).

Nineteen HERB targets intersected the strict set, compared with 8.37 expected (2.27-fold; Fisher odds ratio 2.52). The hypergeometric test (*p* = 6.49 × 10^−4^) and 10,000 expression-matched permutations (*p* = 3.00 × 10^−4^) both supported this overlap (Figure 2B). These 19 genes were taken forward for replication; the broad 81-gene overlap was not enriched above the matched null. Statistical details previously presented in a separate overlap table were consolidated in Figure 2 and its source-data workbook.

### 2.3. Independent Airway-Epithelial Replication and Sputum Description

Twelve of the 19 strict candidates showed the discovery direction and candidate-set BH FDR < 0.05 in GSE67472 (Table 2; Figure 2C). They were *TPSAB1*, *KIT*, *CD44*, *NOS2*, *ADRA2A*, *CHAD*, *NMU*, *FMOD*, *ALPL*, *DDC*, *CTSG*, and *IL1R2*. Eleven also met the genome-wide GSE67472 BH FDR threshold. *IL1R2* met the candidate-set threshold (FDR = 0.0207) but not the genome-wide threshold (FDR = 0.0615).

*TPSAB1* was not measurable in GSE137268 because no valid GPL6104 probe-to-gene mapping for *TPSAB1* was found in either the platform annotation or the processed gene matrix. Ten of the remaining 11 candidates retained the discovery direction, but *IL1R2* was the only gene with same-direction nominal support (*p* = 8.28 × 10^−4^); *NOS2* reversed direction (Figure 2D). These sputum observations were descriptive and did not alter the airway-epithelial candidate set.

### 2.4. Chemical Identity, Herb Sharing, and Evidence Provenance

The 122 exported HERB ingredient records resolved to 113 identity-normalized chemical identities; eight groups contained at least two source records (Supplementary Data, ingredient-identity sheets). Among records linked to the 12 replicated candidates, 26 displayed names resolved to 24 parent-level entities after two further corrections. FER (PubChem CID 445858) and ferulate (CID 54691413) were treated as neutral and deprotonated forms of the same (E)-ferulic-acid parent, while (Z)-ferulic acid remained separate. The aliases geniposide_qt and geniposide were grouped descriptively, without upgrading their evidence status. Records named cis-oleic acid and oleic acid already shared PubChem CID 445639 and the same recorded SMILES, so their raw herb counts were not independent chemical supports.

HERB occurrence counts also showed limited botanical specificity. Before identity consolidation, 18 ingredient records were assigned to one herb, 77 to no more than 10 herbs, and 23 to more than 50 herbs. After consolidation, 16 single-herb identities remained. The shared cis-oleic-acid/oleic-acid identity occurred in more than 50 herb entries. These counts describe the exported database and do not establish chemical uniqueness to *S. ningpoensis*.

Root-specific primary literature supported 10 of the 24 parent-level chemicals at tier A and two at tier B. No verified root-specific source was found for the remaining 12 tier-D identities (Table 3; Supplementary Data, root-occurrence sheet). Tier-A examples included eugenol, (E)-ferulic acid, adenosine, β-sitosterol, aucubin, harpagoside, acteoside, daucosterol, succinic acid, and ursolic acid [6,7,8,9,10,11,16]. Oleic acid and sugiol were assigned to tier B because the recent root annotations were not reported as authentic-standard confirmations [16]. Eleven candidate genes were linked to at least one tier-A or tier-B chemical. *KIT* was linked only to cis-palmitoleic acid, for which root-specific primary evidence was not independently verified.

Ten of the 12 replicated genes had ingredient–target records derived only from database mining. *ADRA2A* also had a herb-level statistical-inference record, but no compound-level target-validation evidence. *NOS2* was the only candidate with a reference-mined regulatory record: acteoside was linked to *NOS2* in experimental autoimmune encephalomyelitis, without a direct binding or target-engagement assay [30]. In a provenance sensitivity analysis, excluding database-mining-only candidates reduced the set from 12 genes to *NOS2* and *ADRA2A*; requiring direct target-engagement evidence retained no gene. Candidate-linked ingredients appeared in 13 GEO series, none involving a human airway-asthma perturbation. One lung series used oleic acid to induce acute injury, and two related series examined brain or blood in the same injury model. Literature-reported root occurrence helped resolve chemical identity but did not validate the imported ingredient–target edges [31].

### 2.5. Integrated Functional Enrichment and STRING Connectivity

**Figure 3:**
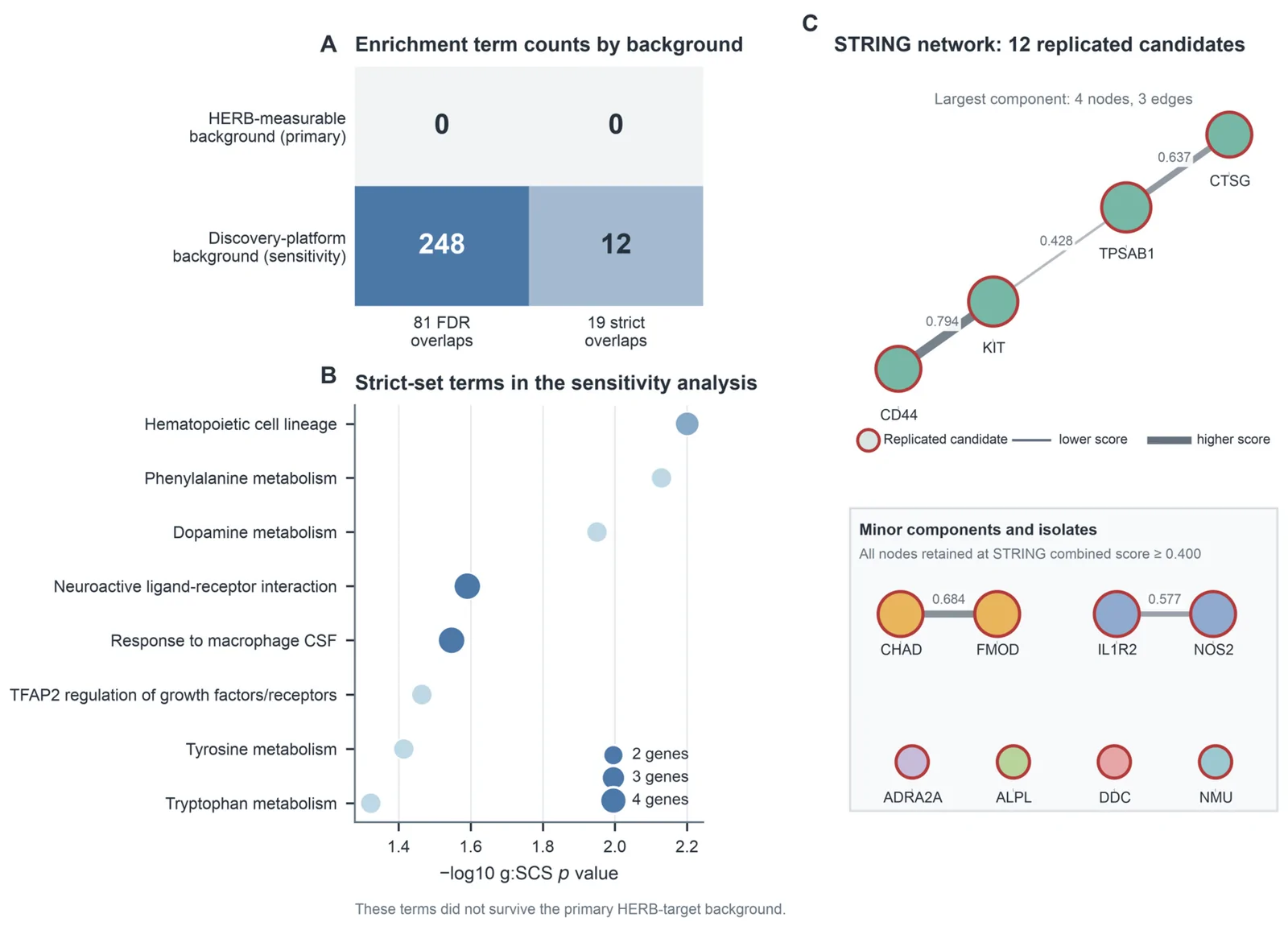
Background-sensitive functional enrichment and sparse STRING connectivity. (**A**) Number of g:SCS-significant terms obtained with the HERB-measurable target background (primary analysis) and the full discovery-platform background (sensitivity analysis). Darker blue shading denotes a larger number of significant terms, whereas gray denotes zero. (**B**) Representative terms for the 19 strict candidates in the platform-background sensitivity analysis; none survived the primary HERB–target background. Bubble size and blue intensity denote the number of intersected genes. (**C**) STRING network for the 12 replicated epithelial candidates at combined score ≥ 0.400. Node-fill colors distinguish connected components, whereas red borders denote replicated-candidate status; component colors do not indicate effect size or rank. Node size denotes degree after square-root scaling, and edge width denotes combined score. All four isolates are retained in the inset.

The integrated enrichment and STRING results are summarized in Figure 3. Neither the 81-gene broad set nor the 19-gene strict set produced a g:SCS-significant term against the 1266 measurable HERB–target background (Figure 3A). Using the full discovery platform as background, the strict set produced 12 terms (Figure 3B), including hematopoietic cell lineage, phenylalanine and tyrosine metabolism, neuroactive ligand–receptor interaction, response to macrophage colony-stimulating factor, and a TFAP2-regulated growth-factor/receptor set. The broad set produced 248 terms with the platform background. Because the prespecified HERB–target analysis was null, these platform-background results were treated as sensitivity findings rather than pathway confirmation.

STRING returned five edges among the 12 replicated candidates: KIT–CD44 (combined score 0.794), CHAD–FMOD (0.684), CTSG–TPSAB1 (0.637), NOS2–IL1R2 (0.577), and KIT–TPSAB1 (0.428). The network comprised seven connected components; the largest contained four proteins, whereas ADRA2A, ALPL, DDC, and NMU were isolated (Figure 3C). Background-sensitive enrichment and sparse connectivity converged on the same interpretation: the candidates did not form a robust, single pathway module.

### 2.6. Nested Model Performance, SHAP Assessment, and Cross-Biospecimen Transfer

**Figure 4:**
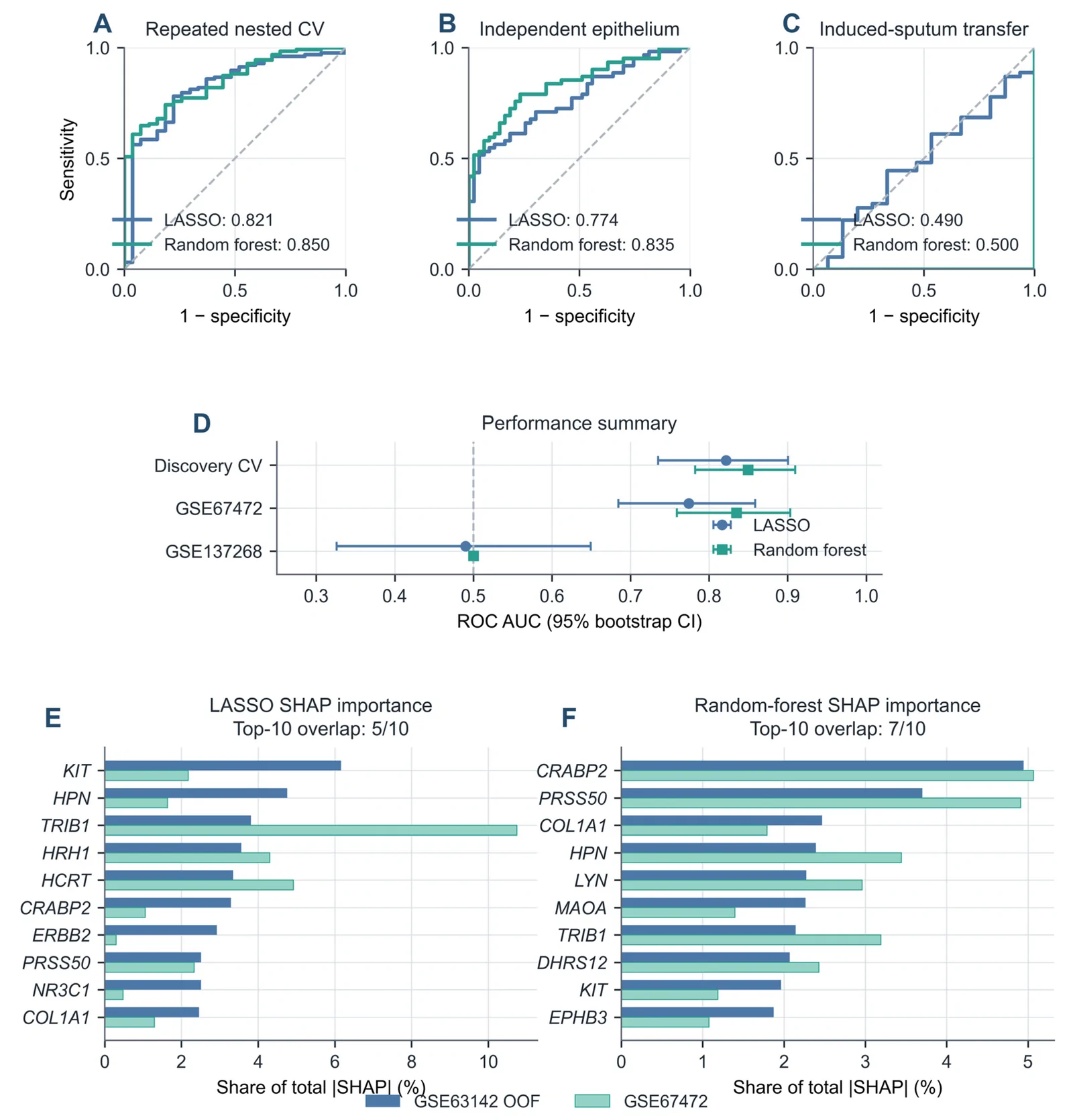
Repeated nested machine-learning performance, external transfer, and model-specific SHAP contributions. (**A**–**C**) ROC curves for L1-penalized logistic regression (LASSO) and random forest using a label-independent feature space of 1163 HERB targets measurable in all three modeled matrices. Feature filtering and model tuning were performed within each outer training fold. GSE43696 was excluded because its subjects overlapped with the discovery cohort. (**D**) ROC AUC estimates and 95% sample-level bootstrap confidence intervals conditional on fixed out-of-fold or ensemble-averaged predictions. The gray dashed diagonals in panels A–C indicate chance/no-discrimination performance; the vertical gray dashed line in panel D marks ROC AUC = 0.5. (**E**,**F**) Global mean absolute SHAP contributions for the ten highest-ranked genes in held-out GSE63142 assessments, normalized within each model to the percentage of total absolute SHAP. LASSO explanations are on the log-odds scale; random-forest explanations are on the class-1 probability scale, so absolute magnitudes are not compared between model classes. SHAP describes fitted prediction contributions and does not establish causal importance, target engagement, herb response, or clinical utility. The original LASSO selection-frequency analysis is provided as Figure S1.

Repeated nested cross-validation in GSE63142 produced ROC AUC 0.821 (95% CI 0.735–0.900) for LASSO and 0.850 (0.782–0.910) for random forest (Table 4; Figure 4). Corresponding PR AUCs were 0.944 and 0.966, against an asthma prevalence of 0.826. In independent GSE67472 airway epithelium, ensemble-averaged predictions yielded ROC AUC 0.774 (0.685–0.859) for LASSO and 0.835 (0.759–0.903) for random forest. Corresponding PR AUCs were 0.860 and 0.899, against a prevalence of 0.590. Conditional on the fixed out-of-fold or ensemble-averaged predictions, all four epithelial ROC AUC permutation *p*-values were 1.00 × 10^−4^.

Transfer to GSE137268 sputum was absent. LASSO ROC AUC was 0.490 (0.326–0.649; permutation *p* = 0.544), while random forest ROC AUC was 0.500 with constant aggregate discrimination (permutation *p* = 1.0). PR AUCs of 0.778 and 0.783 approximated the asthma prevalence of 0.783. Sputum subgroup inspection did not reveal stable rescue: LASSO ROC AUCs were 0.476 for controlled, 0.594 for severe, and 0.444 for uncontrolled asthma, and ranged from 0.212 to 0.635 across inflammatory phenotypes; all corresponding intervals were wide. Random-forest probabilities were constant and yielded ROC AUC 0.500 in every stratum. These negative results cannot distinguish biospecimen composition, phenotype distribution, platform shift, or other cohort effects and therefore do not demonstrate tissue specificity.

SHAP analysis was then applied to the reconstructed outer models, using only the held-out GSE63142 samples for the primary ranking. For LASSO, the ten largest contributors by mean absolute SHAP value were *KIT*, *HPN*, *TRIB1*, *HRH1*, *HCRT*, *CRABP2*, *ERBB2*, *PRSS50*, *NR3C1*, and *COL1A1* (Figure 4E). For random forest, they were *CRABP2*, *PRSS50*, *COL1A1*, *HPN*, *LYN*, *MAOA*, *TRIB1*, *DHRS12*, *KIT*, and *EPHB3* (Figure 4F). These rankings describe contributions to fitted asthma–control predictions within each model class, not biological effect, causality, or herbal target engagement. Absolute SHAP magnitudes were not compared between LASSO and random forest because their explanation scales differed.

When the same fitted models were explained in GSE67472, five of the LASSO top-ten genes and seven of the random-forest top-ten genes overlapped the corresponding discovery out-of-fold lists. Rank correlations across the union of each cohort’s top 50 features were 0.563 for LASSO (*p* = 2.90 × 10^−6^) and 0.693 for random forest (*p* = 5.97 × 10^−10^). These are descriptive cross-cohort rank-stability results, not a second feature-selection or validation step. No sputum SHAP ranking was reported because both models showed near-chance discrimination in GSE137268; interpreting explanations from a non-discriminating transfer model would be uninformative. The original LASSO outer-model selection-frequency analysis is retained as Figure S1. Its most stable features were *KIT* (0.94), *CRABP2* (0.90), *COL1A1* and *PRSS50* (0.70 each), *HRH1* (0.66), *TRIB1* (0.64), and *EPHB3* (0.62).

Excluding *TPSAB1*, *KIT*, *CTSG*, and *IL1R2* where measurable yielded a 1160-feature sensitivity space. Epithelial discrimination remained above chance: LASSO ROC AUC was 0.801 (0.714–0.882) in GSE63142 and 0.753 (0.653–0.842) in GSE67472; random-forest ROC AUC was 0.850 (0.781–0.911) and 0.812 (0.735–0.889), respectively. Sputum transfer remained poor (LASSO ROC AUC 0.298; random forest 0.500). Thus, independent epithelial transfer was not attributable solely to these four prominent markers.

## 3. Discussion

The principal result was a set of 12 airway-brushing-associated genes that met a prespecified replication rule in GSE67472 after a 19-gene strict discovery overlap exceeded the expression-matched expectation in GSE63142. The broader 81-gene overlap was not significant under the matched null. Primary-background enrichment was null, STRING connectivity was sparse, and the models did not transfer to induced sputum. The findings therefore support computational candidate prioritization in two airway-epithelial studies, not a coordinated herbal mechanism.

The study was motivated by the mismatch between limited direct human evidence for *S. ningpoensis* in asthma and the apparent certainty that can arise from database intersections. Several controls materially changed the interpretation. Participant-overlap auditing prevented GSE43696 from being treated as an independent cohort. Expression-matched permutations distinguished the strict overlap from the broad null intersection. Chemical normalization reduced alias duplication, while provenance grading separated prior root occurrence from database target association. GSE67472 then tested epithelial replication, and GSE137268 assessed whether asthma–control prediction transferred across biospecimen types. None of these steps demonstrates pharmacological action.

The cohort audit also limits causal or clinical interpretation. Separate GEO accessions did not guarantee independent participants; 105 of 108 GSE43696 identifiers overlapped GSE63142. GSE67472 was consequently the only independent airway-epithelial replication set available here. Age, sex, phenotype, severity, treatment, inhaled-corticosteroid exposure, and smoking were not uniformly available, so the same adjusted model could not be fitted across studies. No missing covariates were imputed. Residual differences in case mix, treatment, platform, or sample handling may therefore contribute to both replicated and nonreplicated signals.

Botanical and chemical specificity were also limited. Identity normalization reduced 122 ingredient records to 113 chemical identities, and only 16 single-herb identities remained after consolidation. Some identities were widely shared across HERB entries; the cis-oleic-acid/oleic-acid identity, for example, occurred in more than 50 herbs. Root studies supported prior occurrence for selected chemicals, including eugenol, adenosine, acteoside, aucubin, and harpagoside [6,7,8,9,10,11], whereas oleic acid and sugiol remained tentative root assignments [16]. These external observations do not establish the composition of a preparation used in this study because no preparation was analyzed. More importantly, excluding database-mining-only edges reduced the 12 genes to *NOS2* and *ADRA2A*, and a direct-target-engagement requirement retained none. The candidates should therefore be described as genes overlapping HERB annotations rather than as validated or herb-specific targets.

Several replicated genes plausibly capture differences in cellular composition as well as epithelial biology. *NOS2* has an established airway-epithelial context in asthma [32,33], but its direction reversed in sputum. The acteoside–*NOS2* record was reference-mined from experimental autoimmune encephalomyelitis studies and contained no direct binding assay [30]. *TPSAB1*, *KIT*, and *CTSG* may reflect mast-cell or granulocyte contributions; asthma airway brushings can contain intraepithelial mast-cell signals [34,35]. *NMU* has been linked to ILC2-driven allergic lung inflammation in experimental systems [36], but this does not identify the cell producing the present bulk-array signal or show that a Xuanshen constituent acted on that pathway. Bulk profiles cannot distinguish epithelial expression from infiltrating immune or stromal cells [37]. No direct cell-type deconvolution was performed because compatible cell-reference resources and covariates were not uniformly available across these array platforms; the bulk signals were therefore not reassigned to specific cell types.

The enrichment and network analyses did not resolve this heterogeneity into a single module. Terms appeared only when all measured discovery genes were used as the background, not when the prespecified measurable HERB targets were used as the statistical universe. The five STRING edges were distributed across seven components, including four isolated proteins. Platform-background terms and individual edges may inform follow-up experiments, but they are insufficient for a pathway-level mechanism claim.

Machine learning assessed transfer of asthma–control discrimination, not herb response or target importance. Feature filtering and tuning were nested within each outer training fold [28,29]. The SHAP analysis addressed model interpretability without explaining resubstitution predictions: the primary rankings were aggregated only from outer-held-out GSE63142 samples. *KIT* ranked in the top ten for both models and has an airway mast-cell context documented in airway-brushing studies [34,35]. Other recurrent contributors, including *CRABP2*, *HPN*, *TRIB1*, *HRH1*, *PRSS50*, and *COL1A1*, arose from the broader label-independent HERB–target feature space and were not all members of the 12 replicated candidates. SHAP therefore clarifies how the fitted models used measured features, but it neither converts those features into botanical targets nor establishes gene-specific causality [38]. Moderate rank concordance in GSE67472 supports partial transport of the prediction structure, while incomplete top-ten overlap cautions against a fixed mechanistic reading. Excluding four prominent inflammatory or immune-associated markers preserved above-chance discrimination in GSE63142 and GSE67472, suggesting that epithelial transfer was not driven solely by those markers. In contrast, neither pooled sputum nor severity and inflammatory-phenotype strata showed stable discrimination. This negative transfer is compatible with differences in cellular composition, platform, phenotype distribution, processing, or other cohort characteristics [27,39,40]. It cannot establish tissue specificity. PR AUC was considered relative to case prevalence [41], and the external analyses did not assess calibration or fixed clinical thresholds.

Additional limitations remain. The processed datasets were analyzed separately without cross-study batch correction, and public metadata cannot prove participant-level independence for GSE67472. *IL1R2* met the candidate-set but not the genome-wide FDR in GSE67472, and *TPSAB1* could not be evaluated in GSE137268 because it lacked a valid platform mapping. The targeted chemical search was not a systematic review; tier D means that no eligible root-specific source was verified, not that a chemical is absent. No ADME, pharmacokinetic, bioavailability, airway-exposure, target-engagement, or therapeutic-response experiment was performed. Authenticated root material with lot-specific chemical measurement, followed by relevant airway perturbation, orthogonal expression assays, cell-composition assessment, and direct target-engagement tests, would be required before pharmacological or mechanistic claims could be evaluated.

## 4. Materials and Methods

### 4.1. Study Design

This secondary molecular-informatics study used public, de-identified transcriptomic matrices and database records; no botanical material, extract, or preparation was analyzed. The complete workflow is shown in Figure 1. The target analysis started with asthma–control differential expression in airway brushings, followed by HERB–target overlap testing and evaluation of the strict candidates in an independent airway-epithelial dataset. Induced sputum was examined separately to assess transfer across biospecimen types. Chemical-identity, botanical-specificity, and evidence-provenance audits were conducted independently of transcriptomic replication. Machine learning used a label-independent set of HERB targets measurable in all three modeled datasets and was not used to select the replicated candidate set.

In this manuscript, *discovery overlap* refers to the intersection obtained in GSE63142. *Candidate-set replication* required concordant direction and BH correction across the 19 prespecified candidates, whereas *genome-wide replication* also required a dataset-wide BH FDR < 0.05. Directional agreement or classifier performance in sputum was reported as cross-biospecimen transfer evidence and did not count as an additional replication criterion. HERB records were treated as database annotations, without assuming constituent presence, exposure, target engagement, mechanism, or efficacy.

### 4.2. Transcriptome Datasets and Cohort Overlap

Processed series matrices and platform annotations were obtained from the NCBI Gene Expression Omnibus (GEO) [42]. GSE63142 comprised bronchial epithelial samples from 128 participants with asthma and 27 controls, and served as the discovery dataset [43]. GSE43696 comprised 88 asthma and 20 control bronchial epithelial samples on the same GPL6480 platform [32]. Parsing of the short subject identifiers available in GEO metadata showed overlap for 105 of 108 GSE43696 samples with GSE63142. GSE43696 was consequently retained only as a related-cohort descriptive dataset and was excluded from independent replication and model assessment.

GSE67472 contained airway epithelial brushings from 62 participants with asthma and 43 healthy controls on GPL16311 and served as the independent epithelial replication and external machine-learning set [44]. GSE137268 contained induced sputum from 54 asthma and 15 healthy-control samples on GPL6104 and served as the cross-biospecimen transfer set; its GEO record does not list an associated publication [45]. Asthma strata were pooled for the primary asthma–control comparison and were subsequently inspected by clinical-control and inflammatory-phenotype subgroups. Neither dataset shared GEO sample accessions with GSE63142, and their study metadata and biospecimens differed. Participant-level independence could not be verified beyond the available public metadata. The four datasets were analyzed separately, without cross-study merging or batch correction.

### 4.3. Cohort Metadata and Covariate Audit

Age, sex, disease group, severity, inflammatory phenotype, treatment, inhaled-corticosteroid exposure, and smoking were searched in the GEO series matrices, sample characteristics, and associated public metadata. Values were parsed only when a field could be mapped unambiguously; unavailable fields were recorded as missing and were not imputed. Age was summarized by median and interquartile range and categorical fields by counts. Because no common covariate set was available across discovery and external datasets, all primary contrasts remained unadjusted asthma–control comparisons. The complete field-availability matrix and extracted sample-level metadata are provided in the Supplementary Workbook.

### 4.4. HERB Target Records and Provenance Sensitivity

The HERB Scrophulariae Radix entry HERB006280 and its herb–target, ingredient–target, and reference-mining records were parsed from the frozen project export retrieved from HERB (http://herb.ac.cn/; accessed on 18 June 2026) [46]. The export, rather than a later live-database state, was used for reproducibility. Target names were standardized to uppercase human gene symbols, and duplicate targets were collapsed while retaining supporting ingredients and source records. The source table contained 1322 nonredundant targets, of which 1266 were measurable in GSE63142. Herb-level statistical inference, ingredient–target database mining, and reference mining remained separate provenance categories.

Candidate records were assigned to three evidence layers: tier A, direct target-engagement evidence; tier B, nonbinding experimental or statistical evidence beyond database mining; and tier C, database-mining-only association. Sensitivity sets retained all audited candidates, excluded tier-C-only candidates, or required tier-A evidence. These evidence layers concerned the ingredient–target association and were kept separate from the root-occurrence tiers described below.

### 4.5. Ingredient Identity, Herb Sharing, and Supporting Evidence

The frozen HERB006280 export contained 122 ingredient records. Records were consolidated by PubChem CID and then by identical recorded SMILES so that aliases were not counted as separate chemical supports. Records lacking both identifiers remained separate. For candidate-linked ingredients, protonation-state variants were merged when PubChem structures supported a common parent, while stereoisomers remained distinct. Neutral (E)-ferulic acid and ferulate were therefore grouped as one parent, whereas (Z)-ferulic acid was kept separate. Name-only aliases without a CID, including records ending in _qt, were grouped for description but not treated as structure-confirmed equivalents. PubChem was used as the external identifier and structure reference [26]. Counts reported here refer to records in the exported database and do not measure abundance in *S. ningpoensis*. HERB 2.0 informed these normalization rules but did not replace the frozen project export [31].

To examine botanical specificity, the number of HERB entries associated with each ingredient record was retained from the export and summarized before and after chemical-identity consolidation. Single-herb identities and ingredients shared across more than 50 herbs were counted descriptively. This audit measures database sharing, not verified presence or abundance across botanical samples.

A targeted PubMed search for root occurrence was completed through 13 July 2026, with available full texts checked in PubMed Central or on journal websites. Searches combined (“*Scrophularia ningpoensis*” OR “Radix Scrophulariae”), (root OR radix), and each chemical name or alias. Eligible reports were checked for plant part, analytical method, identity claim, and use of authentic standards. Primary studies of *S. ningpoensis* root chemistry were used in preference to reviews or database summaries [6,7,8,9,10,11,12,13,14,15,16]. The supplementary root-occurrence sheet gives the full chemical list, aliases, search decisions, and eligible sources. Tier A required root isolation with structural identification, authentic-standard comparison, or validated quantitation. Tier B indicated a tentative root-profile assignment or identification without authentic-standard confirmation. Tier D indicated that no root-specific primary source was verified in this search; it was not taken to mean that the compound is absent. All occurrence evidence came from previously studied root materials because no botanical sample was analyzed here.

For each replicated gene, we retained the supporting ingredients, normalized herb-occurrence records, evidence category, PubMed identifier, and linked perturbation studies. GEO metadata for candidate-linked experiments were retrieved on 12 July 2026 and classified by organism, tissue, disease context, and the role of the compound as treatment or injury inducer [42]. Reference-mined articles were checked for disease context and direct binding or target-engagement assays. Expression studies involving a compound were used only to describe the perturbation context and did not validate the corresponding HERB ingredient–target record.

No conventional ADME or pharmacokinetic screen was performed. The chemical audit was restricted to identifier normalization, prior root-occurrence evidence, database herb sharing, and ingredient–target provenance; it did not estimate absorption, systemic exposure, airway delivery, metabolism, clearance, or pharmacological plausibility.

### 4.6. Preprocessing and Differential Expression

GEO processed expression values were inspected by range and distribution. All four matrices were already on a log-scale distribution, so no second log transformation was applied. Probe annotations were obtained from the corresponding GEO platform files; GPL16311 identifiers were mapped through NCBI gene information. When multiple probes mapped to one gene, the probe with the largest across-sample variance was retained. This yielded 19,057, 19,553, 18,394, and 17,184 genes for GSE63142, GSE43696, GSE67472, and GSE137268, respectively. *TPSAB1* could not be evaluated in GSE137268 because neither the GPL6104 annotation nor the processed matrix provided a valid *TPSAB1* mapping. Complete preprocessing rules and platform mappings are provided in the Supplementary Workbook.

Asthma-control contrasts were fitted separately in each dataset with limma 3.62.1 using empirical-Bayes moderation, trend = TRUE, and robust = TRUE [47]. *p*-values were adjusted by the Benjamini–Hochberg (BH) method [48]. In discovery, the broad set was defined by BH FDR < 0.05. The strict set additionally required |log2 fold change (log2FC)| ≥ 0.5. No result from GSE43696 contributed to independent candidate counts.

### 4.7. HERB–Target Overlap Tests

For the broad and strict discovery definitions, observed intersections with the 1266 measurable HERB targets were compared with the hypergeometric expectation using the 19,057 tested discovery genes as the universe. Fisher odds ratios were also calculated. To reduce bias from differential detectability, all discovery genes were assigned to average-expression deciles. An empirical null was then generated by repeatedly sampling non-HERB genes to match the decile counts of the measurable HERB target set. Ten thousand permutations were performed with seed 20260712. The empirical *p*-value was calculated as (1 + number of null overlaps at least as large as observed)/10,001.

### 4.8. Airway-Epithelial Replication and Sputum Transfer

The 19 strict discovery candidates were evaluated in GSE67472 without further data-driven expansion. Candidate-set replication required the same log2FC direction as discovery and BH FDR < 0.05 across these 19 tests. Dataset-wide BH FDR values were reported alongside candidate-set values to distinguish targeted replication from genome-wide significance. The replicated candidates were then inspected in GSE137268. Sputum support required both the discovery direction and nominal *p* < 0.05; direction alone was reported descriptively and did not alter the membership of the epithelial candidate set. For model-transfer subgroup inspection, each available clinical-control or inflammatory-phenotype asthma stratum was compared with the same 15 healthy controls. ROC AUC intervals used 2000 stratified bootstrap resamples. These post hoc subgroup results were descriptive because stratum sizes were small and no multiplicity-adjusted hypothesis test was specified.

### 4.9. Functional Enrichment and STRING Analysis

The 81 broad and 19 strict HERB-overlap sets were submitted to g:Profiler (API accessed 12 July 2026) for Gene Ontology biological process, molecular function, and cellular component terms, as well as KEGG, Reactome, and WikiPathways terms [49]. The primary custom background comprised the 1266 measurable HERB targets. The 19,057 discovery genes provided a sensitivity background. Significance was assessed with g:SCS at 0.05. Platform-background terms were reported separately because over-representation results depend on the gene universe and analytical choices [24,25,50].

The candidate-set replicated proteins were queried against STRING 2023 for *Homo sapiens* (taxon 9606) using the full network and a required combined score of 0.400 [51]. All submitted nodes, including proteins without returned associations, were retained. Degree and connected components were calculated from the returned edges. Fixed network coordinates were generated with seed 20260712 and saved with the figure source data. Node degree was used descriptively; it was not combined with expression or machine-learning quantities into a weighted score. Enrichment and network outputs were interpreted together only at the discussion stage; neither analysis was used to redefine the candidate set.

### 4.10. Nested Machine Learning, SHAP Analysis, and Marker-Exclusion Sensitivity

Machine learning assessed asthma-control separation within a database-defined feature space. It did not estimate response to *S. ningpoensis*. The feature universe comprised 1163 HERB targets measurable in GSE63142, GSE67472, and GSE137268 and was defined without class labels. GSE43696 was excluded. Two pipelines were evaluated: standardization, univariate ANOVA filtering, and L1-penalized logistic regression (LASSO) [52]; and univariate filtering followed by a 500-tree random forest with a minimum leaf size of 3 [53]. Class weighting was applied in both models.

GSE63142 was assessed by repeated nested cross-validation: five outer folds repeated ten times and three inner folds for tuning. SelectKBest filtering and hyperparameter selection were fitted only within each outer training split. For LASSO, the StandardScaler was also fitted within the corresponding discovery-training split and then applied unchanged to its held-out fold and the external datasets. Random forest used separately processed, unstandardized expression values. Candidate feature counts were 25, 50, 100, 200, 400, or all features; the LASSO penalty parameter C was searched over 0.01, 0.1, 1, and 10. Keeping feature selection and tuning inside the training folds reduced model-selection and gene-selection bias [28,29]. Each of the 50 fitted outer models generated predictions for GSE67472 and GSE137268; per-sample external probabilities were averaged across these models. Internal probabilities were the mean of ten out-of-fold predictions per sample. Because studies were processed separately and no cross-study harmonization was performed, external performance was interpreted as exploratory transfer under both biological and platform shift [27,40].

Discrimination was summarized by receiver-operating-characteristic area under the curve (ROC AUC) and precision–recall AUC (PR AUC). Ninety-five percent confidence intervals used 2000 stratified sample-level bootstrap resamples of the fixed out-of-fold or ensemble-averaged predictions. ROC AUC permutation *p*-values used 10,000 label permutations against those same fixed predictions; the pipelines were not refitted for each bootstrap or permutation replicate. These quantities therefore represent sample-level uncertainty conditional on the fitted predictions rather than the full uncertainty of model development. PR AUC was interpreted relative to the asthma prevalence of each dataset [41]. Feature stability was the proportion of 50 outer assessments in which a feature remained in the fitted model. Analyses used scikit-learn 1.9.0 [54].

SHAP values were computed after reconstructing the 50 fitted outer models from the recorded split assignments, selected feature sets, and tuned parameters [38]. The primary explanation set comprised only the corresponding outer-held-out GSE63142 samples, thereby avoiding explanation of samples used to fit each model. LASSO was explained with an interventional linear explainer using its outer-training background and was reported on the log-odds scale. Random forest was explained with a tree-path-dependent tree explainer on the class-1 probability scale. For each model and assessment cohort, gene importance was defined as the mean absolute SHAP value across explained samples and outer models, and was normalized to the percentage of total absolute SHAP within that model and cohort. Absolute values were not compared across model classes.

The 50 reconstructed models were also applied unchanged to GSE67472 for a secondary descriptive rank-stability assessment. Cross-cohort stability was summarized by top-ten overlap, Jaccard similarity, and Spearman correlation across the union of the two cohorts’ top 50 features. GSE67472 SHAP rankings were not used for feature selection, model tuning, or candidate definition. No SHAP ranking was reported for GSE137268 because neither model discriminated asthma from controls in that transfer dataset. Reconstructed predictions were required to match the saved predictions, and SHAP additivity was checked for every outer model and assessment cohort.

To assess dependence on prominent inflammatory or immune-associated markers, the complete nested workflow was repeated after excluding *TPSAB1*, *KIT*, *CTSG*, and *IL1R2* wherever measurable. Because *TPSAB1* was already absent from the common three-dataset feature universe, this produced 1160 label-independent features. All folds, tuning rules, external averaging, bootstrap resampling, and permutation procedures were otherwise unchanged.

### 4.11. Reproducibility and Statistical Reporting

All random procedures used seed 20260712. Differential expression was run in R 4.4.3 with limma 3.62.1. The primary workflow used Python 3.12.13, pandas 3.0.3, NumPy 2.5.1, SciPy 1.18.0, scikit-learn 1.9.0, NetworkX 3.6.1, and Matplotlib 3.11.0. SHAP 0.51.0 was run in an isolated clone of that environment with NumPy 2.4.3; scikit-learn and the other model-defining package versions were unchanged. The replay audit reproduced every saved model probability to within 1.12 × 10^−16^, and the largest absolute SHAP additivity error was 5.69 × 10^−14^. Input SHA-256 hashes, preprocessing decisions, cohort-metadata fields, identity-normalized ingredient records, herb-sharing counts, evidence-provenance tiers, cached NCBI metadata, raw enrichment requests and responses, STRING output, fixed coordinates, fold parameters, per-sample predictions, SHAP outputs, marker-exclusion results, sputum subgroup results, and figure source data are provided in the Supplementary Package. Tests were two-sided where applicable. Exact *p*-values are reported except when bounded by the number of permutations.

### 4.12. Use of Generative AI and AI-Assisted Tools

OpenAI Codex was used during manuscript preparation for English-language editing, document-formatting assistance, review and implementation support for reproducible analysis code, and refinement of figure layouts. It was not used to define the research question, select analytical thresholds, generate or alter source data, or make scientific interpretations. All analyses were executed with the deterministic scripts and software versions reported above. The authors checked the code and numerical outputs against the source data, reviewed and edited all AI-assisted material, and take full responsibility for the final content.

## 5. Conclusions

An evidence-audited workflow prioritized 12 asthma airway-brushing-associated genes that overlapped HERB annotations and met the prespecified replication rule in GSE67472. Their interpretation was substantially weakened by broad herb sharing and by the absence of any candidate with direct target-engagement evidence. Null primary-background enrichment, sparse STRING connectivity, and poor sputum transfer further restrict the result to computational prioritization and cross-cohort transferability assessment. The study does not establish the composition, exposure, target engagement, mechanism, or therapeutic activity of *S. ningpoensis*.

## Figures and Tables

**Table 1:** Transcriptome datasets, analytical roles, and cohort overlap.

Dataset (Platform)	Biospecimen; Asthma/Control	Available Participant Characteristics	Analytical Role	Independence Assessment
GSE63142 (GPL6480)	Bronchial epithelial brushing; 128/27	Harmonized age, sex, severity, phenotype, treatment, inhaled-corticosteroid exposure, and smoking unavailable	Discovery	Reference dataset
GSE43696 (GPL6480)	Bronchial epithelial cells; 88/20	Age 35.2 years (IQR 25.7–47.1); 74 female/34 male; 50 moderate and 38 severe asthma cases	Descriptive same-cohort reference only	105/108 short subject identifiers overlap with GSE63142
GSE67472 (GPL16311)	Airway epithelial brushing; 62/43	Age 34 years (IQR 26–41); 54 female/51 male	Independent airway-epithelial replication and external ML test	No shared GEO sample accessions; distinct study metadata
GSE137268 (GPL6104)	Induced sputum; 54/15	Age 61 years (IQR 43–67); 37 female/32 male; 21 controlled, 21 uncontrolled, and 12 severe asthma; phenotype available for 54 samples	Cross-biospecimen transfer and subgroup inspection	No shared GEO sample accessions; distinct biospecimen and study metadata

Note: IQR, interquartile range; ML, machine learning. GSE43696 was not treated as independent because 105 of 108 short subject identifiers overlapped GSE63142. No shared GEO sample accessions and distinct metadata support, but do not prove, participant-level independence for GSE67472 or GSE137268. Harmonized treatment, inhaled-corticosteroid exposure, and smoking variables were unavailable; no missing covariates were imputed.

**Table 2:** Twelve candidates replicated in an independent airway-epithelial study using the discovery-defined candidate set fixed before validation.

Target	GSE63142 log2FC	GSE63142 BH FDR	GSE67472 log2FC	GSE67472 Genome-Wide BH FDR	GSE67472 Candidate-Set BH FDR	GSE137268 log2FC	GSE137268 Nominal *p*
*TPSAB1*	1.234	0.0044	1.056	6.07 × 10^−8^	1.25 × 10^−9^	n.a.	n.a.
*KIT*	0.622	1.10 × 10^−4^	0.691	4.25 × 10^−6^	2.15 × 10^−7^	0.087	0.0830
*CD44*	0.504	0.0028	0.504	1.89 × 10^−5^	1.10 × 10^−6^	0.108	0.2650
*NOS2*	1.104	0.0077	0.443	2.80 × 10^−5^	1.46 × 10^−6^	−0.040	0.1687
*ADRA2A*	0.604	0.0371	0.497	0.0016	3.50 × 10^−4^	0.053	0.3331
*CHAD*	0.533	0.0044	0.314	0.0089	0.0029	0.026	0.7094
*NMU*	0.572	0.0027	0.322	0.0147	0.0049	0.226	0.1713
*FMOD*	0.592	0.0405	0.237	0.0161	0.0049	0.004	0.9041
*ALPL*	0.567	0.0083	0.193	0.0191	0.0055	0.582	0.0733
*DDC*	−0.576	0.0222	−0.092	0.0223	0.0062	−0.033	0.4116
*CTSG*	0.515	0.0305	0.207	0.0360	0.0108	0.012	0.9192
*IL1R2*	0.613	0.0399	0.157	0.0615	0.0207	0.698	8.28 × 10^−4^

Note: Replication required the discovery direction and candidate-set BH FDR < 0.05 in GSE67472. Eleven candidates also met genome-wide GSE67472 BH FDR < 0.05; IL1R2 had genome-wide FDR = 0.0615. n.a., not available because TPSAB1 had no valid GPL6104 probe-to-gene mapping in GSE137268. HERB provenance does not establish direct binding or pharmacological action.

**Table 3:** Root-occurrence evidence and HERB edge provenance for the 12 replicated candidates.

Target	Parent-Level Chemicals, *n*	Representative Support	Highest Root-Occurrence Tier	HERB Edge Provenance and Boundary	Public Perturbation Context
*TPSAB1*	1	eugenol	A: root isolation	Database mining only; occurrence does not validate the eugenol–*TPSAB1* edge	No linked GEO perturbation series
*KIT*	1	cis-palmitoleic acid	D: not independently verified	Database mining only; single-herb occurrence is not chemical confirmation	No linked GEO perturbation series
*CD44*	1	oleic acid	B: tentative root LC-MS annotation	Database mining only; no direct target evidence	6 linked GEO series; 0 human airway/asthma
*NOS2*	18	acteoside and 17 other parent chemicals	A/B/D mixture	Reference-mined acteoside regulation in EAE; no direct binding or target engagement	13 linked GEO series; 0 human airway/asthma
*ADRA2A*	10	eugenol; (E)-ferulic acid; 8 tier-D chemicals	A/D mixture	Herb-level statistical inference plus database mining; no compound-level target validation	4 linked GEO series; 0 human airway/asthma
*CHAD*	1	oleic acid	B: tentative root LC-MS annotation	Database mining only; no direct target evidence	6 linked GEO series; 0 human airway/asthma
*NMU*	1	oleic acid	B: tentative root LC-MS annotation	Database mining only; no direct target evidence	6 linked GEO series; 0 human airway/asthma
*FMOD*	1	oleic acid	B: tentative root LC-MS annotation	Database mining only; no direct target evidence	6 linked GEO series; 0 human airway/asthma
*ALPL*	1	eugenol	A: root isolation	Database mining only; occurrence does not validate the eugenol–*ALPL* edge	No linked GEO perturbation series
*DDC*	1	adenosine	A: root isolation	Database mining only; occurrence does not validate the adenosine–*DDC* edge	No linked GEO perturbation series
*CTSG*	2	eugenol; oleic acid	A/B mixture	Database mining only; no direct target evidence	6 linked GEO series; 0 human airway/asthma
*IL1R2*	1	eugenol	A: root isolation	Database mining only; occurrence does not validate the eugenol–*IL1R2* edge	No linked GEO perturbation series

Note: Tier A denotes prior root isolation/structural identification, authentic-standard confirmation, or validated quantitation; tier B denotes root detection with tentative mass-spectrometric assignment; tier D denotes no independently verified root-specific primary evidence in the targeted search. These tiers concern prior botanical occurrence only. No constituent was measured in a study preparation, and no literature-occurrence tier was treated as evidence of binding, target engagement, exposure, or efficacy.

**Table 4:** Repeated nested cross-validation and external-transfer performance.

Model	Assessment	*n*	ROC AUC (95% CI)	PR AUC (95% CI)	Permutation *p*
LASSO	GSE63142 repeated nested CV	155	0.821 (0.735–0.900)	0.944 (0.900–0.978)	1.00 × 10^−4^
LASSO	GSE67472 independent epithelium	105	0.774 (0.685–0.859)	0.860 (0.801–0.913)	1.00 × 10^−4^
LASSO	GSE137268 induced sputum	69	0.490 (0.326–0.649)	0.778 (0.704–0.883)	0.5441
Random forest	GSE63142 repeated nested CV	155	0.850 (0.782–0.910)	0.966 (0.950–0.980)	1.00 × 10^−4^
Random forest	GSE67472 independent epithelium	105	0.835 (0.759–0.903)	0.899 (0.851–0.942)	1.00 × 10^−4^
Random forest	GSE137268 induced sputum	69	0.500 (0.500–0.500)	0.783 (0.783–0.783)	1.0000

Note: The feature universe comprised 1163 label-independent HERB targets. Confidence intervals used 2000 stratified sample-level bootstrap resamples of the fixed out-of-fold or ensemble-averaged predictions; permutation *p*-values used 10,000 label permutations against those fixed predictions. The intervals and *p*-values, therefore, do not include uncertainty from repeating model development. PR AUC should be interpreted relative to case prevalence.

## Data Availability

Expression data are available from GEO under GSE63142, GSE43696, GSE67472, and GSE137268. Database provenance was obtained from the frozen HERB entry HERB006280 export. Analysis scripts, identity-normalized ingredient tables, cached NCBI metadata, derived statistical tables, fixed network coordinates, per-sample model predictions, SHAP values and audit summaries, and figure source data are supplied as Supporting Files. No unreported patient-level data were generated.

## Supplementary Materials

The following supporting information can be downloaded at: https://www.mdpi.com/article/10.3390/ijms27177886/s1.
