# Supplementary material for: Cross-Cohort Transcriptomic Prioritization and Chemical-Provenance Assessment of Asthma Airway-Brushing Genes Overlapping HERB-Annotated Scrophularia ningpoensis Targets
Source: Int J Mol Sci. 2026 Sep 3;27(17):7886. doi: 10.3390/ijms27177886 (PMC13566514; doi:10.3390/ijms27177886)
Supplement: Supplementary file 1 [file ijms-27-07886-s001.zip › figures.zip!/Supplementary_Figures_Revised_2026-08-21.pdf]

Figure S1. Stability of the most frequently selected LASSO genes

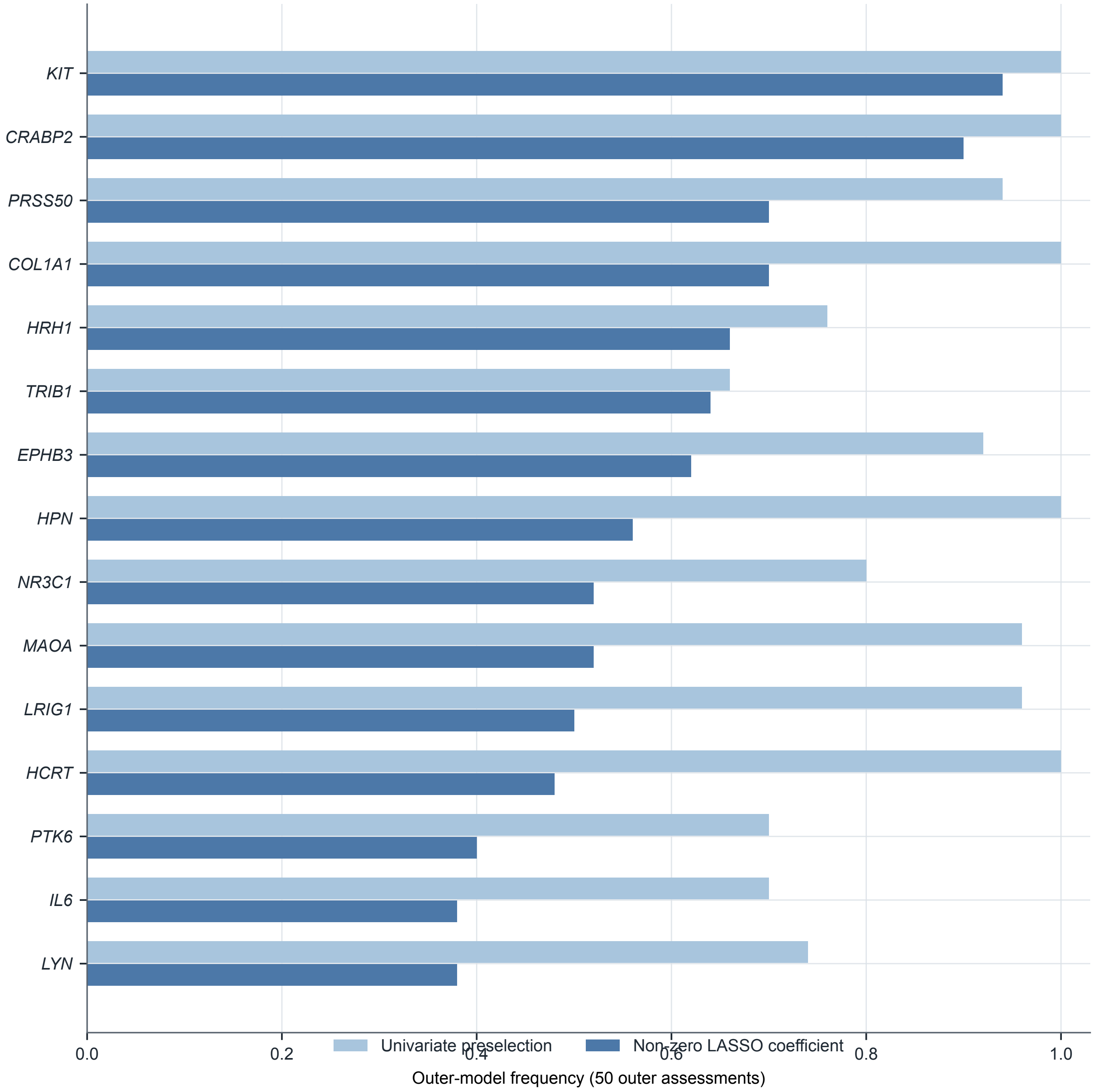

Figure S1. Stability of the 15 genes most frequently assigned non-zero coefficients by LASSO across the 50 outer assessment models (five-fold outer cross-validation repeated ten times). Pale bars show the frequency with which a gene entered the within-fold univariate preselection set; dark bars show the frequency with which the fitted LASSO retained a non-zero coefficient. The analysis evaluates selection stability and is distinct from the SHAP attribution analysis shown in Figure 4E.

Data source: ml_feature_stability_2026-07-12.tsv. No model was refitted for this display.
